# A high-precision and high-order error model for airborne distributed POS transfer alignment

**DOI:** 10.1038/s41598-020-77595-w

**Published:** 2020-11-24

**Authors:** Bin Gu, Wen Ye, Zijie Teng, Hongmei Chen, Guochen Wang

**Affiliations:** 1 China Academy of Electronics and Information Technology, Beijing, 100041 China; 2 National Institute of Metrology, Beijing, 100029 China; 3 Beijing Institute of Metrology, Beijing, 100029 China; 4 School of Electrical Engineering, He’nan University of Technology, Zhengzhou, 450001 China; 5 Harbin Institute of Technology, Harbin, 150001 China

**Keywords:** Aerospace engineering, Electrical and electronic engineering

## Abstract

Distributed position and orientation systems (DPOSs) can provide abundant time-spatial information for interferometric synthetic aperture radar (InSAR) in airborne earth observation systems. However, some key error terms have not been taken into consideration in the traditional low-order error model, which suppresses the performance of the slave POS and further cannot meet the compensation precision of InSAR. To improve the compensation precision, a precise high-order error model with 45 dimensions was derived. Not only does it take into account the influence of scale factor errors and installation errors of the gyro and accelerometer, but it also makes use of random constants and a first-order Markov process model to describe the gyro drift and accelerometer bias. In addition, the flexure angle and its angular rate were added to the state variables of the transfer alignment model. Based on the model, a measurement equation for attitude errors that considers flexure was deduced. Then, a transfer alignment model based on the matching algorithm including position-velocity-attitude was designed. Finally, the proposed model was validated by simulated and real tests, and the experimental results show that its performance is obviously better than that of the traditional model.

## Introduction

Position and orientation systems, as a kind of INS/GPS integrated inertial navigation system in nature, can provide successive, abundant, highly precise and high-frequency time-spatial information for remote sensing loads^1,2^. With the rapid development of airborne observation technology, high-resolution and high-efficiency stereoscopic imaging with multitask imaging sensors or interferometric synthetic aperture radar (InSAR) has become the most attractive development direction of aerial surveys and remote sensing systems and urgently demands a distributed position and orientation system (DPOS) to accurately measure multinode time-spatial reference information to carry out motion compensation for imaging.

**Figure 1:**
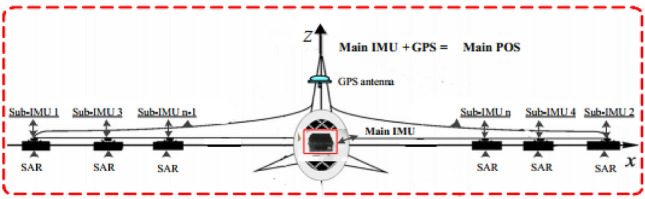
DPOS installation layout.

DPOSs are composed of a high-precision main POS, a few low-precision slave POSs, a computer system and postprocessing software. The main POS integrates a main inertial measurement unit (IMU) and a global positioning system (GPS)^2,3^. As shown in Fig. 1, in general, the main POS is installed on the belly, which is also called the main node. Slave POSs are installed on the wing as close as possible to the load, which are also called subnodes and consist only of sub-IMUs. The main POS provides high-precision motion parameters for each slave POS as reference information, which can obtain motion information of each subsystem with transfer alignment technology^1,2^ and furthermore carry out motion compensation for InSAR.

Due to the wing deformation caused by the external disturbance, there is a time-varying lever between the multinodes that seriously degrades the performance of transfer alignment and correspondingly affects the baseline measurement accuracy. Therefore, the traditional transfer alignment model cannot meet the requirements of some high-precision interferometric imaging tasks. To solve this problem, some researchers have carried out studies that focus on error models and matching methods.

DPOS errors include the estimation error and the measurement errors of the IMU and GPS. Among them, the main POS errors have been widely studied. The measurement errors of the IMU are the main factors affecting POS accuracy. Usually, the calibration factor and installation errors (nonorthogonality errors between gyro and accelerometer) of the IMU still exist after calibration compensation, so residual calibration error must be fully considered in error modeling. At present, POS/AV610 produced by the Applanix Company of Canada has the highest accuracy. It adopts a high-order error model based on many errors; the real-time horizontal attitude accuracy reaches 0.005° and the real-time heading accuracy reaches 0.02° under the condition of real-time differential GPS. In^4^, a 28-dimensional error model is established. The calibration factors of gyros and accelerometers and the residual installation error are taken into account. However, the random drift of gyros and the random bias of accelerometers are simply taken as random constant values, which is not suitable for DPOSs. In^5^, a 36-dimensional error model is proposed. The random constant, random walk and first-order Markov process are used to represent the random drift of the gyro and the random bias of the accelerometer. In addition, the residual installation error is ignored. These simplifications directly affect the accuracy of the DPOS. In high-precision aerial remote sensing motion compensation, the influence of residual calibration and other factors must be fully considered, so it is necessary to establish an accurate high-order error model. On the other hand, the measurement errors of the slave IMU with low precision are the main factors that affect the accuracy of the DPOS. The scale factor and installation errors of the IMU are still in the residual errors after calibration, so the calibration errors must be considered in modeling the errors^6^.

The attitude-velocity matching model is used in the 24-dimensional transfer alignment model^7,8^. The model is simple and easily applied, but scale factors and installation errors are not considered. Moreover, the position information is not contained in the matching model. Therefore, the baseline accuracy between these two systems is low^9^. Although the traditional attitude-velocity matching model can significantly improve the attitude and velocity accuracy, the accuracy of the position and baseline of the subnodes directly affect the resolution of the InSAR when the DPOS is applied to InSAR. Therefore, the typical application of the DPOS requires a high-accuracy baseline, which is more urgent for ultrahigh-resolution aerial surveys and remote sensing^10,11^. In this application, it is urgent to obtain the high-precision position information of the subnodes.

Therefore, in this paper, considering more error terms, a high-precision and high-order transfer alignment model with 45 dimensions is derived. Then, in order to provide more high-precision position information and improve the baseline accuracy, a matching algorithm including position-velocity-attitude is proposed. On this basis, the method is verified by simulation experiments and vehicle and flight tests.

The organization of this paper is as follows. “Introduction” presents the work related to the proposed methodology. “Error model description” presents an error model description. “Modeling of transfer alignment for a distributed POS” describes the transfer alignment model for a DPOS. “Experimental results” discusses the analysis of the simulation and real tests. Finally, “Conclusions” provides a conclusion.

## Error model description

The accuracy of IMU in POS is affected by many error sources, mainly including gyro drift, acceleration bias, scale factor errors, installation errors and so on. We first list the error model of the IMU.

Due to the low accuracy of the slave IMU, the corresponding error parameters must be calibrated^12–14^. The error model can be described as follows.

1 $$ \left\{ {\begin{array}{*{20}c} {\delta \omega = \varepsilon + \left( {\delta K_{g} + \delta G} \right) \times \omega_{ib}^{b} } \\ {\delta f = \nabla + \left( {\delta K_{a} + \delta A} \right) \times f^{b} } \\ \end{array} } \right. $$

where $\delta \omega$ and $\delta f$ are the gyro and accelerometer errors, respectively, and $\varepsilon$ and $\nabla$ are the random drift of the gyro and random bias of acceleration, respectively. In addition, $\delta K_{g}$ and $\delta G$ are the scale factor errors of the gyro and accelerometer, respectively, $\delta K_{a}$ and $\delta A$ are the installation errors of the gyro and accelerometer, respectively, and $\omega_{ib}^{b}$ and $f^{b}$ are the inputs of the gyro and accelerometer, respectively.

### The gyro drift and accelerometer bias

Gyro drift and accelerometer bias reflect the errors of the angular rate and acceleration, including random constants, first-order Markov processes, and white noise^15,16^.

2 $$ \left\{ \begin{gathered} \varepsilon (t) = \varepsilon_{g} (t) + \varepsilon_{m} (t) + \omega_{g} (t) \hfill \\ \nabla (t) = \nabla_{a} (t) + \nabla_{m} (t) + \omega_{a} (t) \hfill \\ \end{gathered} \right. $$

where $\varepsilon_{g} (t)$, $\varepsilon_{m} (t)$, $\omega_{g} (t)$ and $\nabla_{a} (t)$, $\nabla_{m} (t)$, $\omega_{a} (t)$ represent the gyro and accelerometer random constants, first-order Markov processes, and white noise, respectively. The mathematical description is as follows.

3 $$ \dot{\varepsilon }_{g} = 0,\;\dot{\varepsilon }_{m} = - \frac{1}{\alpha }\varepsilon_{m} + w_{g} $$

4 $$ E[w_{g} (t)w_{g} (\tau )] = q_{g} \delta (t - \tau ) $$

5 $$ \dot{\nabla }_{a} = 0,\;\dot{\nabla }_{m} = - \frac{1}{\beta }\nabla_{m} + w_{a} $$

6 $$ E[w_{a} (t)w_{a} (\tau )] = q_{a} \delta (t - \tau ) $$

where $\alpha$ and $\beta$ are the related times of the first-order Markov process of the gyroscope and the accelerometer, respectively; $w_{g}$ and $w_{a}$ are the white noise of the first-order Markov process; $q_{g}$ and $q_{a}$ are the white noise intensity; and $\delta (t - \tau )$ is the Dirac function.

### Scale factor error of the gyro and accelerometer

Because the output of the IMU consists of pulse signals, the corresponding angular rate and acceleration must be calculated according to certain ratio coefficients. The coefficients are obtained by calibration, but deviation exists between the calibration value and the actual value, which is called the scale factor error. Its mathematical description can be expressed as follows:

7 $$ \delta \dot{K}_{g} = 0,\;\delta \dot{K}_{a} = 0 $$

where $\delta K_{g} = diag[\delta K_{gx} ,\delta K_{gy} ,\delta K_{gz} ]$ and $\delta K_{a} = diag[\delta K_{ax} ,\delta K_{ay} ,\delta K_{az} ]$ are the scale factor errors of the gyro and accelerometer, respectively.

### Installation error of the gyro and accelerometer

The three axes of gyroscopes and accelerometers are not orthogonal due to the installation relationship, and the error is the installation error^17^, which can be described mathematically as follows:

8 $$ \delta \dot{G} = 0,\;\delta \dot{A} = 0 $$

where $$\delta G = \left[ {\begin{array}{*{20}c} 0 & { - G_{yz} } & {G_{zy} } \\ {G_{xz} } & 0 & { - G_{zx} } \\ { - G_{xy} } & {G_{yx} } & 0 \\ \end{array} } \right]$$ and $$\delta A = \left[ {\begin{array}{*{20}c} 0 & { - A_{yz} } & {A_{zy} } \\ {A_{xz} } & 0 & { - A_{zx} } \\ { - A_{xy} } & {A_{yx} } & 0 \\ \end{array} } \right]$$ are the installation error matrices of the gyro and accelerometer, respectively.

In addition, the overall error model $\delta \omega$ and $\delta f$ of the gyroscope and accelerometer can be described by the following equation.

9 $$\left\{ {\begin{array}{ll} {\delta \omega = \varepsilon_{b} + \varepsilon_{m} + \omega_{g} + (\delta K_{g} + \delta G) \times \omega_{ib}^{b} } \\ {\delta f = \nabla_{b} + \nabla_{m} + \omega_{a} + (\delta K_{a} + \delta A) \times f_{{}}^{b} } \\ \end{array} } \right.$$

### The flexural motion equation of the wing

To describe the flexible deformation angle more accurately, a two-order Markov process is used as the flexible deformation angle model^18^. Moreover, the three axial flexible deformation processes are assumed to be independent. Therefore, the three axial flexible deformation angle models can be obtained as follows:

10 $$ \ddot{\theta }_{x} + 2\beta_{x} \dot{\theta }_{x} + \beta_{x}^{2} \theta_{x}^{{}} = \eta_{x} $$

11 $$ \ddot{\theta }_{y} + 2\beta_{y} \dot{\theta }_{y} + \beta_{y}^{2} \theta_{y}^{{}} = \eta_{y} $$

12 $$ \ddot{\theta }_{z} + 2\beta_{z} \dot{\theta }_{z} + \beta_{z}^{2} \theta_{z}^{{}} = \eta_{z} $$

where $\beta_{i} = \frac{2.146}{{\tau_{i} }}(i = x,y,z)$; $\theta_{x}$, $\theta_{y}$, and $\theta_{z}$ are flexible deformation angles; $\tau_{i}$ is the related time corresponding to the flexible deformation angle; and $\eta_{x} ,\eta_{y} ,\eta_{z}$ represents white noise with variance:

13 $$ Q_{i} = 4\beta_{i}^{3} \sigma_{i}^{2} \, (i = x,y,z) $$

## Modeling of transfer alignment for a distributed POS

On the basis of “Error model description”, the error model of the IMU, the traditional position, velocity, and attitude error model can be integrated as follows.

Position error equation:

14 $$ \left\{ {\begin{array}{*{20}l} {\delta \dot{L} = - \frac{{V_{N} \cdot \delta h}}{{(R_{m} + h)^{2} }} + \frac{{\delta V_{N} }}{{R_{n} + h}}} \\ {\delta \dot{\lambda } = \frac{{V_{E} \cdot \sec L \cdot \tan L \cdot \delta L}}{{R_{n} + h}} - \frac{{V_{E} \cdot \sec L \cdot \delta h}}{{(R_{n} + h)^{2} }} + \frac{{\sec L \cdot \delta V_{E} }}{{R_{n} + h}}} \\ {\delta \dot{h} = \delta V_{U} } \\ \end{array} } \right. $$

Velocity error equation:

15 $$ \begin{gathered} \hfill \\ \left\{ {\begin{array}{*{20}l} \begin{gathered} \delta \dot{V}_{E} = f_{N} \phi_{U} - f_{U} \phi_{N} + (\frac{{V_{N} \tan L - V_{U} }}{{R_{n} + h}})\delta V_{E} + (2\omega_{ie} \sin L + \frac{{V_{E} \tan L}}{{R_{n} + h}})\delta V_{N} + (2\omega_{ie} V_{N} \cos L + \frac{{V_{E} V_{N} \sec^{2} L}}{{R_{n} + h}} + 2\omega_{ie} V_{U} \sin L)\delta L - (2\omega_{ie} \cos L + \frac{{V_{E} }}{{R_{n} + h}})\delta V_{U} \hfill \\ + \frac{{V_{E} V_{U} - V_{E} V_{N} \tan L}}{{(R_{n} + h)^{2} }}\delta h + \nabla_{E} + C_{11} (f_{x}^{s} \delta K_{Ax} { + }f_{y}^{s} \delta A_{xz} - f_{z}^{s} \delta A_{xy} ) + C_{12} (f_{y}^{s} \delta K_{Ay} - f_{x}^{s} \delta A_{yz} + f_{z}^{s} \delta A_{yx} ) + C_{13} (f_{z}^{s} \delta K_{Az} + f_{x}^{s} \delta A_{zy} - f_{y}^{s} \delta A_{zx} ) \hfill \\ \end{gathered} \hfill \\ \begin{gathered} \delta \dot{V}_{N} = f_{U} \phi_{E} - f_{E} \phi_{U} - 2(\omega_{ie} \sin L + \frac{{V_{E} \tan L}}{{R_{n} + h}})\delta V_{E} - \frac{{V_{U} \delta V_{N} }}{{R_{m} + h}} - \frac{{V_{N} \delta V_{U} }}{{R_{m} + h}} - (2\omega_{ie} \cos L + \frac{{V_{E} \sec^{2} L}}{{R_{n} + h}})V_{E} \delta L + \frac{{V_{N} V_{U} + V_{E} V_{E} \tan L}}{{(R_{n} + h)^{2} }}\delta h + \hfill \\ \, \nabla_{N} + C_{21} (f_{x}^{s} \delta K_{Ax} { + }f_{y}^{s} \delta A_{xz} - f_{z}^{s} \delta A_{xy} ) + C_{22} (f_{y}^{s} \delta K_{Ay} - f_{x}^{s} \delta A_{yz} + f_{z}^{s} \delta A_{yx} ) + C_{23} (f_{z}^{s} \delta K_{Az} + f_{x}^{s} \delta A_{zy} - f_{y}^{s} \delta A_{zx} ) \hfill \\ \end{gathered} \hfill \\ \begin{gathered} \delta \dot{V}_{U} = - f_{N} \phi_{E} + f_{E} \phi_{N} + 2(\omega_{ie} \cos L + \frac{{V_{E} }}{{R_{n} + h}})\delta V_{E} + 2\frac{{V_{N} \delta V_{N} }}{{R_{m} + h}} - 2\omega_{ie} V_{E} \sin L\delta L - \frac{{V_{E} V_{E} + V_{N} V_{N} }}{{(R_{n} + h)^{2} }}\delta h + \nabla_{U} \hfill \\ \, + C_{31} (f_{x}^{s} \delta K_{Ax} { + }f_{y}^{s} \delta A_{xz} - f_{z}^{s} \delta A_{xy} ) + C_{32} (f_{y}^{s} \delta K_{Ay} - f_{x}^{s} \delta A_{yz} + f_{z}^{s} \delta A_{yx} ) + C_{33} (f_{z}^{s} \delta K_{Az} + f_{x}^{s} \delta A_{zy} - f_{y}^{s} \delta A_{zx} ) \hfill \\ \end{gathered} \hfill \\ \end{array} } \right. \hfill \\ \end{gathered} $$

Attitude error equation:

16 $$ \left\{ {\begin{array}{*{20}l} \begin{aligned} \dot{\phi }_{E} &= - \frac{{\delta V_{N} }}{{R_{m} + h}} + (\omega_{ie} \sin L + \frac{{V_{E} \tan L}}{{R_{n} + h}})\phi_{N} - (\omega_{ie} \cos L + \frac{{V_{E} }}{{R_{n} + h}})\phi_{U} + \frac{{V_{N} }}{{(R_{m} + h)^{2} }}\delta h - \varepsilon_{E} \\&\quad - C_{11} (\omega_{ibx}^{s} \delta K_{Gx} - \omega_{ibz}^{s} \delta G_{xy} + \omega_{iby}^{s} \delta G_{xz} ) - C_{12} (\omega_{iby}^{s} \delta K_{Gy} - \omega_{ibx}^{s} \delta G_{yz} + \omega_{ibz}^{s} \delta G_{yx} ) + C_{13} (\omega_{ibz}^{s} \delta K_{Gz} + \omega_{ibx}^{s} \delta G_{zy} + \omega_{iby}^{s} \delta G_{zx} ) \\ \end{aligned}  \hfill \\ \begin{aligned} \dot{\phi }_{N} &= \frac{{\delta V_{E} }}{{R_{n} + h}} - \omega_{ie} \sin L\delta L - (\omega_{ie} \sin L + \frac{{V_{E} \tan L}}{{R_{n} + h}})\phi_{E} - \frac{{V_{N} }}{{R_{m} + h}}\phi_{U} - \frac{{V_{E} }}{{(R_{n} + h)^{2} }}\delta h - \varepsilon_{N} \\&\quad - C_{21} (\omega_{ibx}^{s} \delta K_{Gx} - \omega_{ibz}^{s} \delta G_{xy} + \omega_{iby}^{s} \delta G_{xz} ) - C_{22} (\omega_{iby}^{s} \delta K_{Gy} - \omega_{ibx}^{s} \delta G_{yz} + \omega_{ibz}^{s} \delta G_{yx} ) - C_{23} (\omega_{ibz}^{s} \delta K_{Gz} + \omega_{ibx}^{s} \delta G_{zy} + \omega_{iby}^{s} \delta G_{zx} ) \\ \end{aligned} \hfill \\ \begin{aligned} \dot{\phi }_{U} &= \frac{{\tan L\delta V_{E} }}{{R_{n} + h}} + (\omega_{ie} \cos L + \frac{{V_{E} \sec^{2} L}}{{R_{n} + h}})\delta L + (\omega_{ie} \cos L + \frac{{V_{E} }}{{R_{n} + h}})\phi_{E} + \frac{{V_{N} \phi_{N} }}{{R_{m} + h}} - \frac{{V_{E} \tan L\delta h}}{{(R_{n} + h)^{2} }} - \varepsilon_{U} \hfill \\&\quad \, - C_{31} (\omega_{ibx}^{s} \delta K_{Gx} - \omega_{ibz}^{s} \delta G_{xy} + \omega_{iby}^{s} \delta G_{xz} ) - C_{32} (\omega_{iby}^{s} \delta K_{Gy} - \omega_{ibx}^{s} \delta G_{yz} + \omega_{ibz}^{s} \delta G_{yx} ) - C_{33} (\omega_{ibz}^{s} \delta K_{Gz} + \omega_{ibx}^{s} \delta G_{zy} + \omega_{iby}^{s} \delta G_{zx} ) \hfill \\ \end{aligned} \hfill \\ \end{array} } \right. $$

where $R_{n}$ and $R_{m}$ are the main curvature radii of the vertical circle and meridian, respectively; $f^{b}$ is the output of the accelerometer in the body coordinate frame, $\omega_{ie}^{n}$ is the angular velocity of the earth, $\omega_{en}^{n}$ is the angular velocity of displacement, $\omega_{ib}^{s}$ is the output of the gyro in the slave IMU coordinate frame, and $C_{b}^{n}$ denotes the transformation matrix between the body frame and the navigation frame.

Furthermore, considering the flexible deformation model of the wing, the traditional 15-order error model can be presented as the following high-order model; then, the state space model description is given as^19–22^:

17 $$ \left\{ {\begin{array}{*{20}l} {\dot{X} = FX + Gw} \hfill \\ {Z = HX + v} \hfill \\ \end{array} } \right. $$

where $X \in R^{45}$ is the system state vector, $F \in R^{45 \times 45}$ is the dynamic coefficient matrix, $G \in R^{45 \times 9}$ is the dynamic noise distribution matrix, and $w \in R^{9}$ is a zero-mean white-noise vector. The state vector $X$ includes attitude errors $\phi_{E}$,$\phi_{N}$,$\phi_{U}$; velocity errors $\delta V_{E}$, $\delta V_{N}$, $\delta V_{U}$; position errors $\delta L$,$\delta \lambda$,$\delta h$; gyro random constant drifts $\varepsilon_{gx}$,$\varepsilon_{gy}$,$\varepsilon_{gz}$; gyro first-order Markov process models $\varepsilon_{mx}$,$\varepsilon_{my}$,$\varepsilon_{mz}$; gyro scale factor errors $\delta K_{Gx}$,$\delta K_{Gy}$,$\delta K_{Gz}$; gyro installation errors $\delta G_{yz}$,$\delta G_{zy}$,$\delta G_{xz}$,$\delta G_{zx}$,$\delta G_{xy}$, $\delta G_{yx}$; accelerometer random constants $\nabla_{ax}$ ,$\nabla_{ay}$ ,$\nabla_{az}$; accelerometer first-order Markov process models $\nabla_{mx}$, $\nabla_{my}$, $\nabla_{mz}$; accelerometer scale factor errors $\delta K_{Ax}$, $\delta K_{Ay}$, $\delta K_{Az}$; accelerometer installation errors $\delta A_{yz}$, $\delta A_{zy}$, $\delta A_{xz}$, $\delta A_{zx}$, $\delta A_{xy}$, $\delta A_{yx}$; flexure deformation angle $\theta_{x}$,$\theta_{y}$,$\theta_{z}$; and flexure deformation angle rate $\dot{\theta }_{x}$,$\dot{\theta }_{y}$,$\dot{\theta }_{z}$.

The dynamic coefficient matrix $F$ is expressed as follows:

$$ F = \left[ \begin{gathered} \begin{array}{*{20}c} {F_{INS} } & {F_{s1} } & {0_{9 \times 3} } & {0_{9 \times 9} } & {F_{s2} } & {0_{9 \times 3} } & {0_{9 \times 9} } & {0_{9 \times 3} \begin{array}{*{20}c} {} & {0_{9 \times 3} } \\ \end{array} } \\ {0_{3 \times 9} } & {0_{3 \times 3} } & {0_{3 \times 3} } & {0_{3 \times 9} } & {0_{3 \times 3} } & {0_{3 \times 3} } & {0_{3 \times 9} } & {0_{3 \times 3} \begin{array}{*{20}c} {} & {0_{3 \times 3} } \\ \end{array} } \\ {0_{3 \times 9} } & {0_{3 \times 3} } & {P_{1} } & {0_{3 \times 9} } & {0_{3 \times 3} } & {0_{3 \times 3} } & {0_{3 \times 9} } & {0_{3 \times 3} \begin{array}{*{20}c} {} & {0_{3 \times 3} } \\ \end{array} } \\ {0_{9 \times 9} } & {0_{9 \times 3} } & {0_{9 \times 3} } & {0_{9 \times 9} } & {0_{9 \times 3} } & {0_{9 \times 3} } & {0_{9 \times 9} } & {0_{9 \times 3} \begin{array}{*{20}c} {} & {0_{9 \times 3} } \\ \end{array} } \\ {0_{3 \times 9} } & {0_{3 \times 3} } & {0_{3 \times 3} } & {0_{3 \times 9} } & {0_{3 \times 3} } & {0_{3 \times 3} } & {0_{3 \times 9} } & {0_{3 \times 3} \begin{array}{*{20}c} {} & {0_{3 \times 3} } \\ \end{array} } \\ {0_{3 \times 9} } & {0_{3 \times 3} } & {0_{3 \times 3} } & {0_{3 \times 9} } & {0_{3 \times 3} } & {P_{2} } & {0_{3 \times 9} } & {0_{3 \times 3} \begin{array}{*{20}c} {} & {0_{3 \times 3} } \\ \end{array} } \\ {0_{9 \times 9} } & {0_{9 \times 3} } & {0_{9 \times 3} } & {0_{9 \times 9} } & {0_{9 \times 3} } & {0_{9 \times 3} } & {0_{9 \times 9} } & {0_{9 \times 3} \begin{array}{*{20}c} {} & {0_{9 \times 3} } \\ \end{array} } \\ {0_{3 \times 9} } & {0_{3 \times 3} } & {0_{3 \times 3} } & {0_{3 \times 9} } & {0_{3 \times 3} } & {0_{3 \times 3} } & {0_{3 \times 9} } & {0_{3 \times 3} \begin{array}{*{20}c} {} & {I_{3} } \\ \end{array} } \\ \end{array} \hfill \\ \begin{array}{*{20}c} {0_{3 \times 9} } & {0_{3 \times 3} } & {0_{3 \times 3} } & {0_{3 \times 9} } & {0_{3 \times 3} } & {0_{3 \times 3} } & {0_{3 \times 9} } & {\begin{array}{*{20}c} {} \\ \end{array} B_{1} \begin{array}{*{20}c} {} & {B_{2} } \\ \end{array} } \\ \end{array} \hfill \\ \end{gathered} \right] $$

$$ F_{INS} = \left[ {\begin{array}{*{20}c} {F_{1} } & {F_{2} } & {F_{3} } \\ {F_{4} } & {F_{4} } & {F_{5} } \\ {F_{1} } & {F_{2} } & {0_{3 \times 3} } \\ \end{array} } \right] $$

$$ F_{1} = \left[ {\begin{array}{*{20}c} 0 & {\omega_{ie} \sin L + \frac{{V_{x} \tan L}}{{R_{n} + h}}} & { - (\omega_{ie} \cos L + \frac{{V_{x} }}{{R_{n} + h}})} \\ { - (\omega_{ie} \sin L + \frac{{V_{x} \tan L}}{{R_{n} + h}})} & 0 & { - \frac{{V_{y} }}{{R_{m} + h}}} \\ {\omega_{ie} \cos L + \frac{{V_{x} }}{{R_{n} + h}}} & {\frac{{V_{y} }}{{R_{m} + h}}} & 0 \\ \end{array} } \right] \cdot F_{2} = \left[ {\begin{array}{*{20}c} 0 & { - \frac{1}{{R_{m} + h}}} & 0 \\ {\frac{1}{{R_{n} + h}}} & 0 & 0 \\ {\frac{\tan L}{{R_{m} + h}}} & 0 & 0 \\ \end{array} } \right] $$

$$ F_{3} = \left[ {\begin{array}{*{20}c} 0 & 0 & {\frac{{V_{y} }}{{(R_{m} + h)^{2} }}} \\ { - \omega_{ie} \sin L} & 0 & { - \frac{{V_{x} }}{{(R_{n} + h)^{2} }}} \\ {\omega_{ie} \cos L + \frac{{V_{x} \sec^{2} L}}{{R_{n} + h}}} & 0 & {\frac{{V_{x} \tan L}}{{(R_{n} + h)^{2} }}} \\ \end{array} } \right] \cdot F_{4} = \left[ {\begin{array}{*{20}c} 0 & { - f_{z} } & {f_{y} } \\ {f_{z} } & 0 & { - f_{x} } \\ { - f_{y} } & {f_{x} } & 0 \\ \end{array} } \right] $$

$$ F_{5} = \left[ {\begin{array}{*{20}c} {\frac{{V_{y} \tan L}}{{R_{n} + h}} - \frac{{V_{z} }}{{R_{n} + h}}} & {2\omega_{ie} \sin L + \frac{{V_{x} \tan L}}{{R_{n} + h}}} & { - 2\omega_{ie} \cos L - \frac{{V_{x} }}{{R_{n} + h}}} \\ { - 2(\omega_{ie} \sin L + \frac{{V_{x} \tan L}}{{R_{n} + h}})} & { - \frac{{V_{z} }}{{R_{m} + h}}} & { - \frac{{V_{y} }}{{R_{m} + h}}} \\ {2(\omega_{ie} \cos L + \frac{{V_{x} }}{{R_{n} + h}})} & {\frac{{2V_{y} }}{{R_{m} + h}}} & 0 \\ \end{array} } \right] $$

$$ F_{6} = \left[ {\begin{array}{*{20}c} {2\omega_{ie} V_{y} \cos L + \frac{{V_{x} V_{y} \sec^{2} L}}{{R_{n} + h}} + 2\omega_{ie} V_{z} \sin L} & 0 & {\frac{{V_{x} V_{z} - V_{x} V_{y} \tan L}}{{(R_{n} + h)^{2} }}} \\ { - {(2}\omega_{ie} \cos L + \frac{{V_{x} \sec^{2} L}}{{R_{n} + h}}{)}V_{x} } & 0 & {\frac{{V_{y} V_{z} + V_{x}^{2} \tan L}}{{(R_{n} + h)^{2} }}} \\ { - 2\omega_{ie} V_{x} \sin L} & 0 & { - \frac{{V_{x}^{2} + V_{y}^{2} }}{{(R_{n} + h)^{2} }}} \\ \end{array} } \right] $$

$$ F_{7} = \left[ {\begin{array}{*{20}c} 0 & 0 & { - \frac{{V_{y} }}{{(R_{m} + h)^{2} }}} \\ {\frac{{V_{x} \sec L\tan L}}{{R_{n} + h}}} & 0 & { - \frac{{V_{x} \sec L}}{{(R_{n} + h)^{2} }}} \\ 0 & 0 & 0 \\ \end{array} } \right] \cdots F_{8} = \left[ {\begin{array}{*{20}c} 0 & {\frac{1}{{R_{m} + h}}} & 0 \\ {\frac{\sec L}{{R_{n} + h}}} & 0 & 0 \\ 0 & 0 & 1 \\ \end{array} } \right] $$

$$ F_{s1} = \left[ {\begin{array}{*{20}c} {0_{6 \times 3} } \\ {C_{b}^{n} } \\ \end{array} } \right] \cdot F_{s2} = \left[ {\begin{array}{*{20}c} {0_{3 \times 3} } \\ {C_{b}^{n} } \\ {0_{3 \times 3} } \\ \end{array} } \right] $$

$$ P_{1} = \left[ {\begin{array}{*{20}c} { - \frac{1}{{\alpha_{x} }}} & 0 & 0 \\ 0 & { - \frac{1}{{\alpha_{y} }}} & 0 \\ 0 & 0 & { - \frac{1}{{\alpha_{z} }}} \\ \end{array} } \right] \cdot P_{2} = \left[ {\begin{array}{*{20}c} { - \frac{1}{{\beta_{x} }}} & 0 & 0 \\ 0 & { - \frac{1}{{\beta_{y} }}} & 0 \\ 0 & 0 & { - \frac{1}{{\beta_{z} }}} \\ \end{array} } \right] $$

$$ B_{1} = \left[ {\begin{array}{*{20}c} { - \eta_{x}^{2} } & 0 & 0 \\ 0 & { - \eta_{y}^{2} } & 0 \\ 0 & 0 & { - \eta_{z}^{2} } \\ \end{array} } \right] \cdot B_{2} = \left[ {\begin{array}{*{20}c} { - 2\eta_{x}^{{}} } & 0 & 0 \\ 0 & { - 2\eta_{y}^{{}} } & 0 \\ 0 & 0 & { - 2\eta_{z}^{{}} } \\ \end{array} } \right] $$

The dynamic noise distribution matrix ${\varvec{G}}$ can be expressed by:

$$ {\varvec{G}} = \left[ {\begin{array}{*{20}c} {C_{b}^{n} } & {0_{3 \times 3} } & {0_{3 \times 3} } \\ {0_{3 \times 3} } & {C_{b}^{n} } & {0_{3 \times 3} } \\ {0_{36 \times 3} } & {0_{36 \times 3} } & {0_{36 \times 3} } \\ {0_{3 \times 3} } & {0_{3 \times 3} } & {I_{3 \times 3} } \\ \end{array} } \right] $$

The dynamic disturbance noise $w$ can be described by:

18 $$ {\varvec{w}} = \left[ {\begin{array}{*{20}c} {w_{{\varepsilon_{x} }} } & {w_{{\varepsilon_{y} }} } & {w_{{\varepsilon_{z} }} } \\ \end{array} \begin{array}{*{20}l} {w_{{\nabla_{x} }} } \hfill & {w_{{\nabla_{y} }} } \hfill & {w_{{\nabla_{z} }} } \hfill & {w_{\theta x} } \hfill & {w_{\theta y} } \hfill & {w_{\theta z} } \hfill \\ \end{array} } \right]^{T} $$

${\varvec{Z}}$ is the measurement vector, ${\varvec{H}}$ is the measurement matrix, and $v$ is the measurement noise. The measurement matrix can be expressed as:

$$ {\varvec{H}} = \left[ {\begin{array}{*{20}c} {H_{2} } & {0_{3 \times 3} } & {0_{3 \times 3} } & {0_{3 \times 15} } & {0_{3 \times 15} } & {H_{3} } & {0_{3 \times 3} } \\ {0_{3 \times 3} } & {I_{3} } & {0_{3 \times 3} } & {0_{3 \times 15} } & {0_{3 \times 15} } & {0_{3 \times 3} } & {0_{3 \times 3} } \\ {0_{3 \times 3} } & {0_{3 \times 3} } & {H_{1} } & {0_{3 \times 15} } & {0_{3 \times 15} } & {0_{3 \times 3} } & {0_{3 \times 3} } \\ \end{array} } \right] $$

$$ H_{1} = \left[ {\begin{array}{*{20}c} {R_{m} + h} & 0 & 0 \\ 0 & {(R_{n} + h)\cos L} & 0 \\ 0 & 0 & 1 \\ \end{array} } \right] $$

$$ H_{2} = \left[ {\begin{array}{*{20}c} {\frac{{T_{a}^{(12)} T_{a}^{(32)} }}{{(T_{a}^{(12)} )^{2} + (T_{a}^{(22)} )^{2} }}} & 0 & { - 1} \\ { - \frac{{T_{a}^{(22)} }}{{\sqrt {1 - (T_{a}^{(32)} )^{2} } }}} & {\frac{{T_{a}^{(12)} }}{{\sqrt {1 - (T_{a}^{(32)} )^{2} } }}} & 0 \\ {\frac{{T_{a}^{(21)} T_{a}^{(33)} - T_{a}^{(31)} T_{a}^{(23)} }}{{(T_{a}^{(33)} )^{2} + (T_{a}^{(31)} )^{2} }}} & {\frac{{T_{a}^{(31)} T_{a}^{(13)} - T_{a}^{(11)} T_{a}^{(33)} }}{{(T_{a}^{(33)} )^{2} + (T_{a}^{(31)} )^{2} }}} & 0 \\ \end{array} } \right] $$

$$ H_{3} = \left[ {\begin{array}{*{20}c} {\frac{{T_{a}^{(12)} T_{a}^{(23)} - T_{a}^{(13)} T_{a}^{(22)} }}{{(T_{a}^{(12)} )^{2} + (T_{a}^{(22)} )^{2} }}} & 0 & {\frac{{T_{a}^{(11)} T_{a}^{(22)} - T_{a}^{(12)} T_{a}^{(21)} }}{{(T_{a}^{(12)} )^{2} + (T_{a}^{(22)} )^{2} }}} \\ {\frac{{T_{a}^{(33)} }}{{\sqrt {1 - (T_{a}^{(32)} )^{2} } }}} & 0 & { - \frac{{T_{a}^{(31)} }}{{\sqrt {1 - (T_{a}^{(32)} )^{2} } }}} \\ { - \frac{{T_{a}^{(31)} T_{a}^{(32)} }}{{(T_{a}^{(33)} )^{2} + (T_{a}^{(31)} )^{2} }}} & 1 & { - \frac{{T_{a}^{(32)} T_{a}^{(33)} }}{{(T_{a}^{(33)} )^{2} + (T_{a}^{(31)} )^{2} }}} \\ \end{array} } \right] $$

where $T_{a}$ is the attitude matrix of the main POS and where the superscript $(ij)$ denotes the $i$ th row and $j$ th column element of $T_{a}$.

## Experimental results

### Physical simulation

To verify the feasibility of the established flexural motion models in this paper, the “S + U” trajectory is used for simulation.

Before the simulation, the position, velocity and attitude parameters of the “S + U” trajectory are generated by the trajectory generator, and the theoretical data of the gyro and accelerometer are also produced. The measurement noise of the main POS is superimposed on the position, velocity and attitude of the trajectory data as the real output. Constant and random noise of the slave POS are superimposed on the theoretical value of the gyros and accelerometers, which are converted through the deflection angle.

The main POS utilizes a high-precision IMU. The measurement accuracies of the header, pitch and roll are 0.005° (1 $\sigma$), 0.002° (1 $\sigma$) and 0.002° (1 $\sigma$), respectively. The measurement noise of the speed is 0.005 m/s (1 $\sigma$). The measurement noise of the latitude, longitude, and height is 0.05 m (1 $\sigma$), 0.05 m (1 $\sigma$), and 0.05 m (1 $\sigma$), respectively. The gyro constant and random drift of the slave POS are 0.01°/h, and the accelerometer constant and random bias are 20 μg. The strapdown computation cycle of the slave IMU is 0.01 s, and the filter cycle is 0.05 s.

#### The flight track

**Figure 2:**
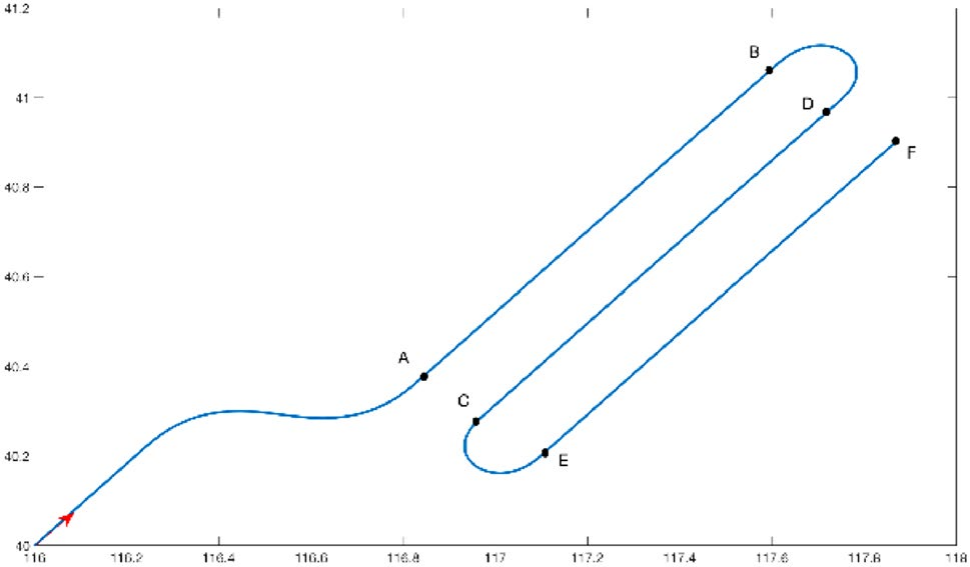
Flight trajectory.

The AB segment, CD segment and EF segment are uniform linear flight segments in Fig. 2. The total flight time is 4500 s, among which the earlier 900 s is the S trajectory. The U trajectory is the test time. The AB segment and CD segment can be regarded as imaging segments. The BD segment is the maneuver turn. The initial longitude position of the aircraft is 116°E, and the latitude is 40°N. The initial azimuth angle, pitch angle and roll angle are 45°, 0° and 0°, respectively. The flight speed is 100 m/s.

#### The flight process

The velocity and height remain constant throughout the flight process, and the roll angle is taken into account at maneuvering corners. Specific flight process settings are shown in Table 1

**Table 1:** The flight process setting.

Time (s)	Movement mode
0–900	“S” trajectory
900–1900	imaging segment
1900–2200	Maneuver turn
2200–3200	imaging segment
3200–3500	Maneuver turn
3500–4500	imaging segment

**Figure 3:**
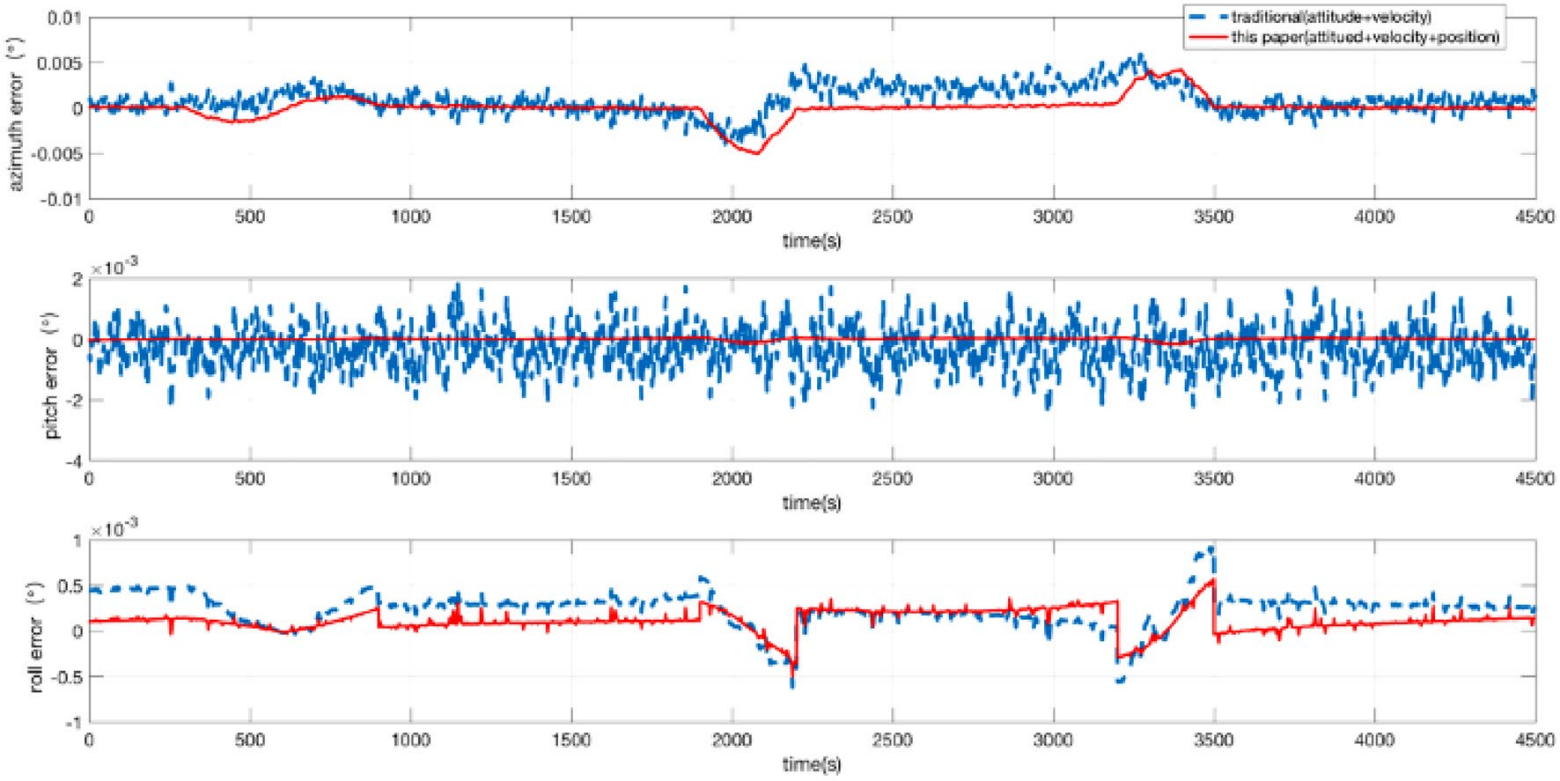
Attitude error of the POS.

**Figure 4:**
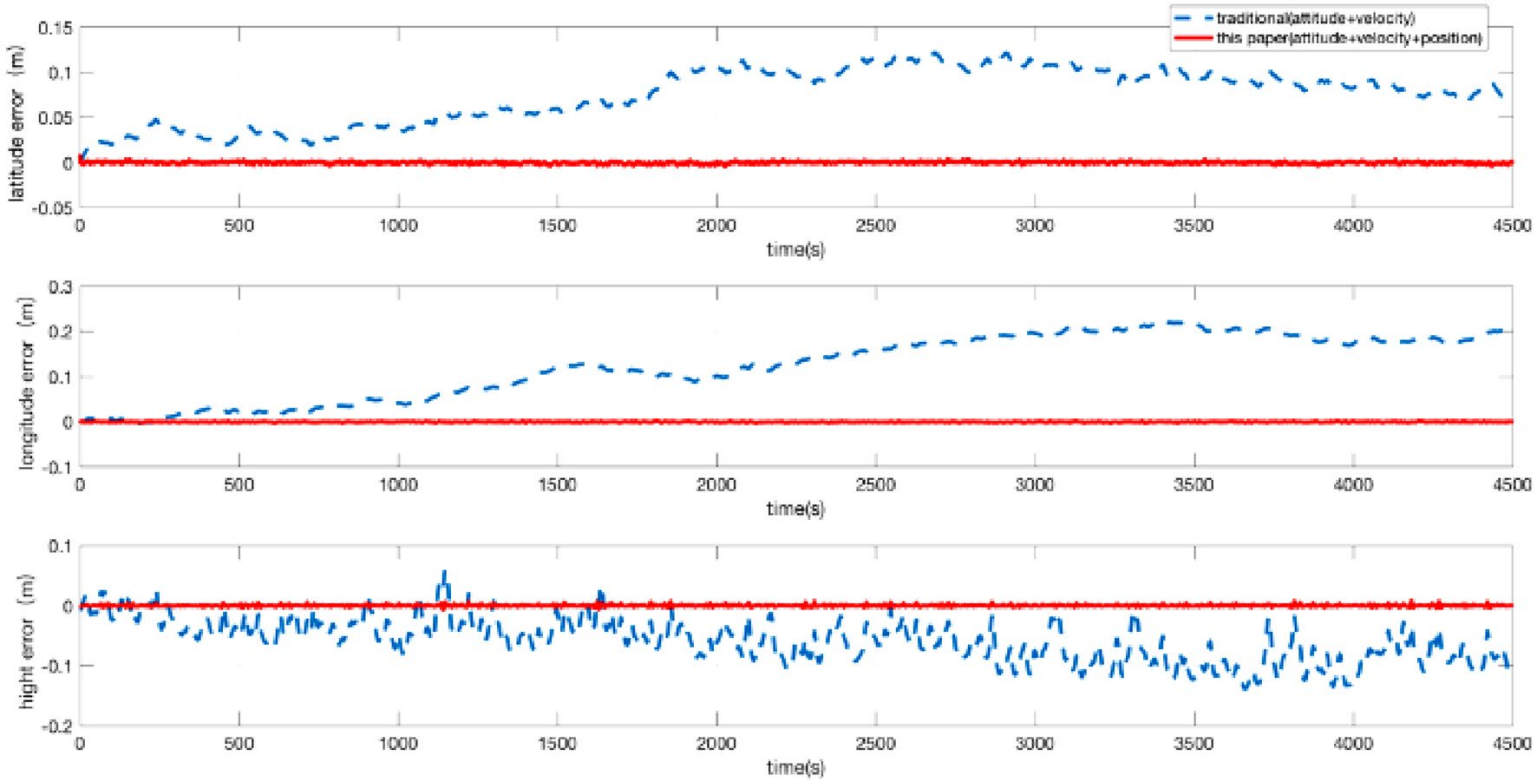
Position error of the POS.

The position + velocity + attitude matching method is used in this paper. To verify the accuracy and the necessity of the 45-dimensional high-order error models, the accuracy of the navigation results is compared with that of the low-dimensional error models that use the “attitude + velocity” matching method. Then, the attitude, speed, and position error are given in Figs. 3 and 4. The accuracy of the position is improved by several times. The accuracy of attitude is slightly increased. The accuracy of the azimuth angle is improved by 24.24%. The accuracy of pitch angle is improved by 44.44%. The accuracy of the roll angle is improved by 53.57%. A comparison of the experimental results is shown in Table 2.

**Table 2:** The relative navigation errors for the imaging segment (STD).

Standard deviation (STD)	Traditional error model	Error model in this paper
**Attitude error (°)**		
Azimuth	0.00165	0.00125
Pitch	0.00072	0.00040
Roll	0.00028	0.00013
**Position error (m)**		
Latitude	0.03056	0.00103
Longitude	0.07201	0.00081
Height	0.05713	0.00175

### Vehicle experiment

**Figure 5:**
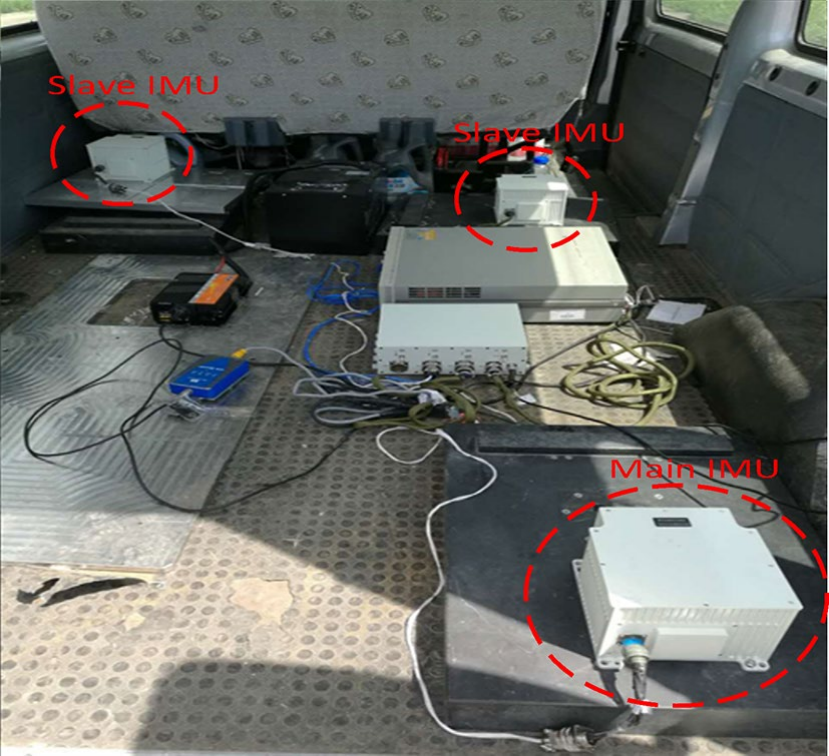
The land vehicle experiment.

**Figure 6:**
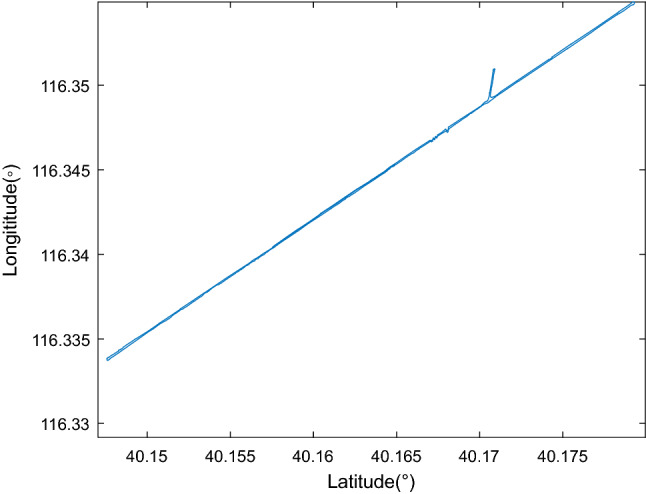
The vehicle trajectory.

To validate the precision of the error models by the proposed methods in this paper, vehicle experiments are carried out. Three IMUs and different GPS navigation equipment are fixed in an experimental vehicle, as shown in Fig. 5, and the trajectory is shown in Fig. 6. One of them is the main IMU, and the other two are slave IMUs. The trajectory is a part of the Fourth Ring Road of Beijing.

**Figure 7:**
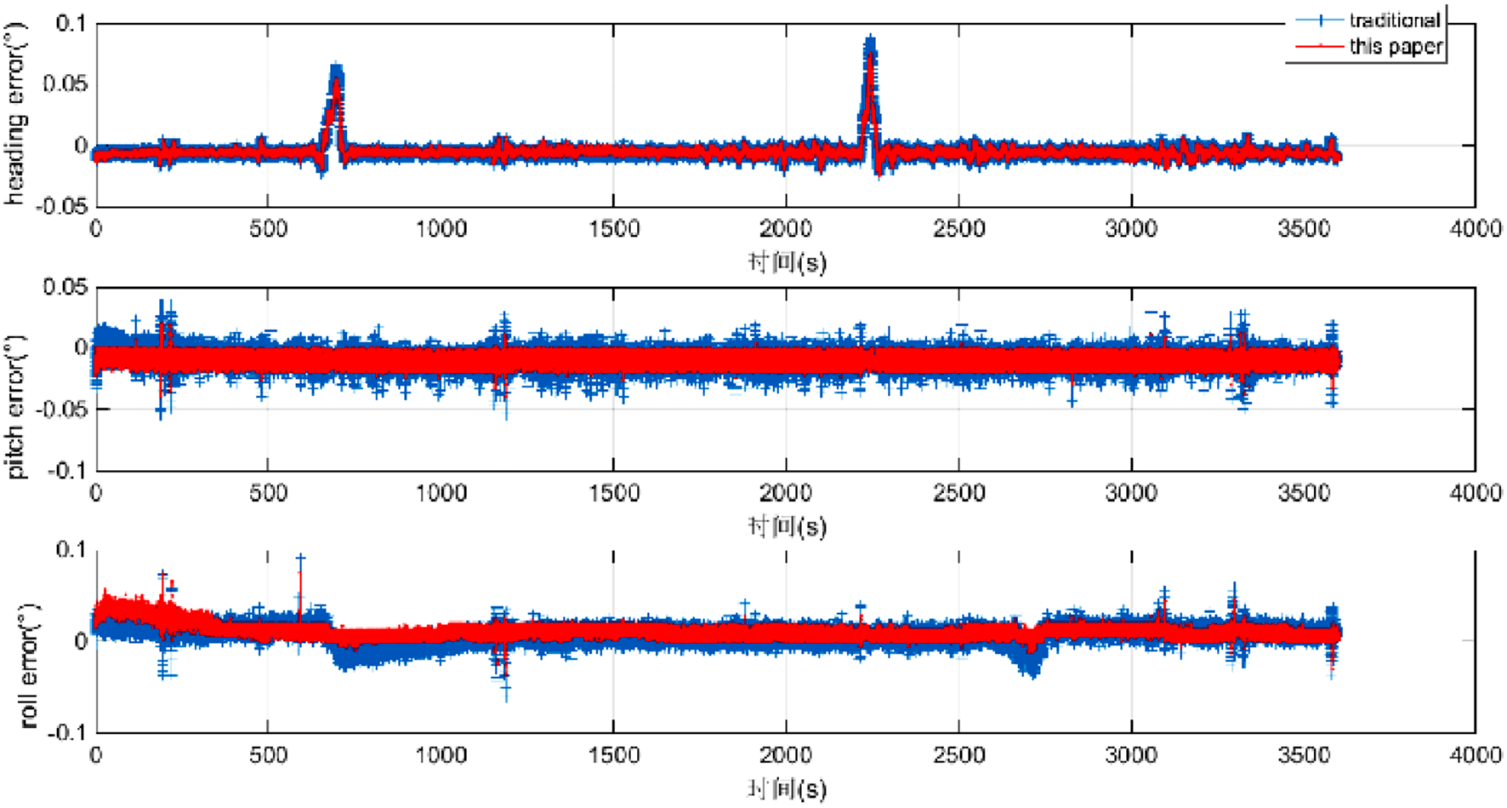
Attitude error comparison.

**Figure 8:**
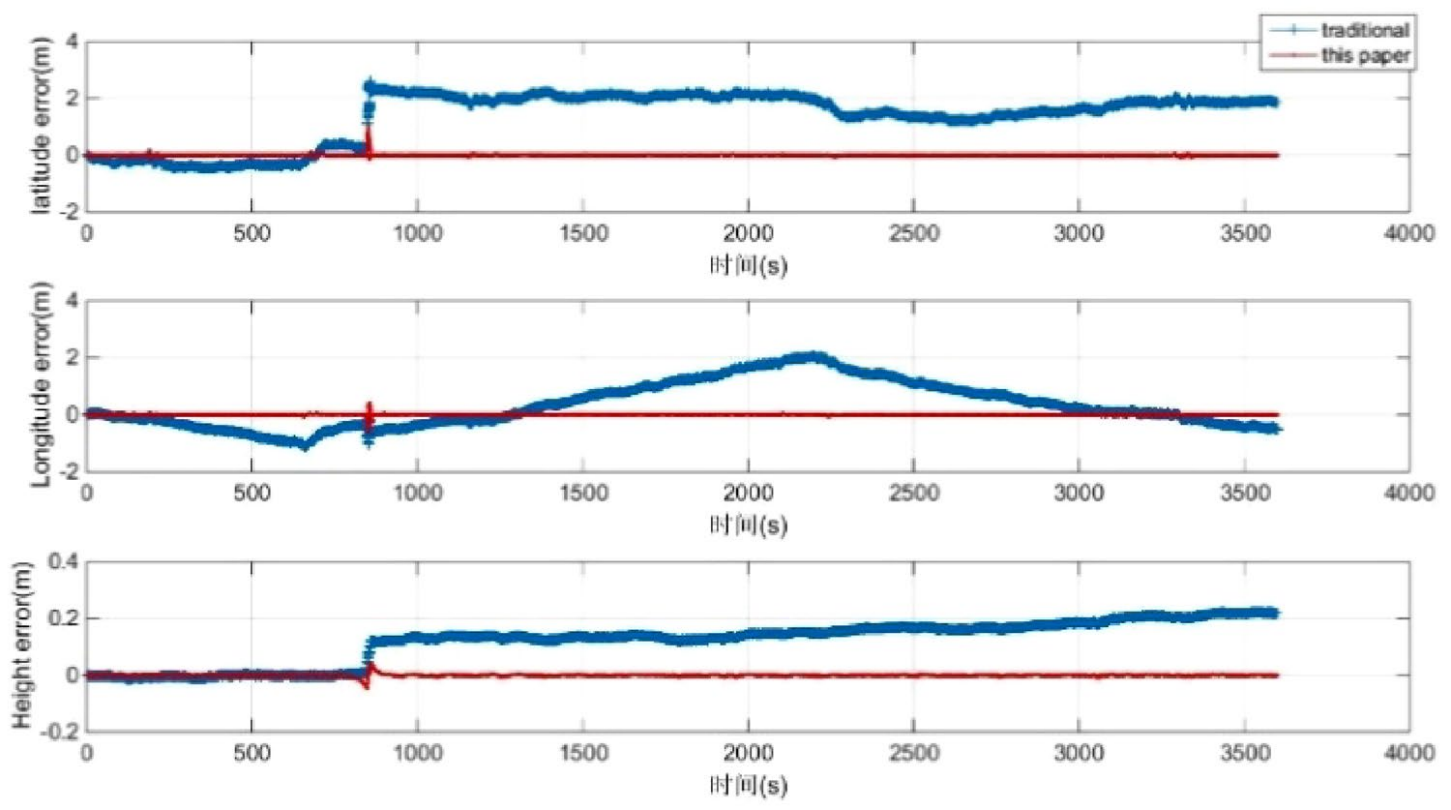
Position error comparison.

First, the vehicle is parked in a certain location for 300 s, and the data are sampled for initial alignment. Then, the vehicle is driven along the layout route for more than 3600 s. The differential GPS navigation positions are regarded as reference marks during the vehicle experiments. Finally, the smooth results of the integrated navigation of different GPSs and IMUs are used as the benchmarks to compare the errors of the models proposed in this paper and the traditional models, as shown in Figs. 7 and 8. Table 3 shows that the position accuracy has been obviously improved, and the accuracy of the azimuth angle, pitch angle and roll angle are increased by 21.61%, 26.27%, and 35.73%, respectively.

**Table 3:** The relative navigation errors for the imaging segment (STD).

Standard deviation (STD)	Traditional error model	Error model in this paper
**Attitude error (°)**		
Azimuth	0.00273	0.00214
Pitch	0.00628	0.00463
Roll	0.00750	0.00482
**Position error (m)**		
Latitude	0.42166	0.04181
Longitude	0.80979	0.02780
Height	0.03081	0.00856

### Flight experiment

**Figure 9:**
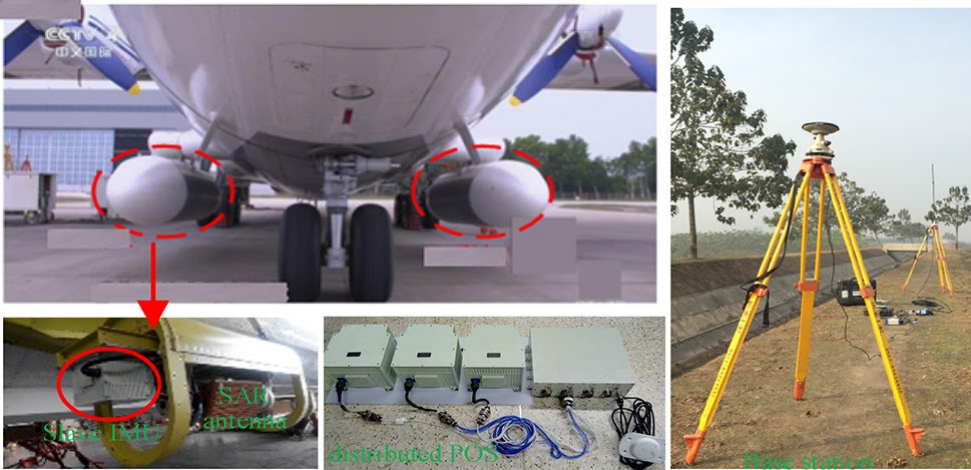
Experiment airplane and DPOS.

**Figure 10:**
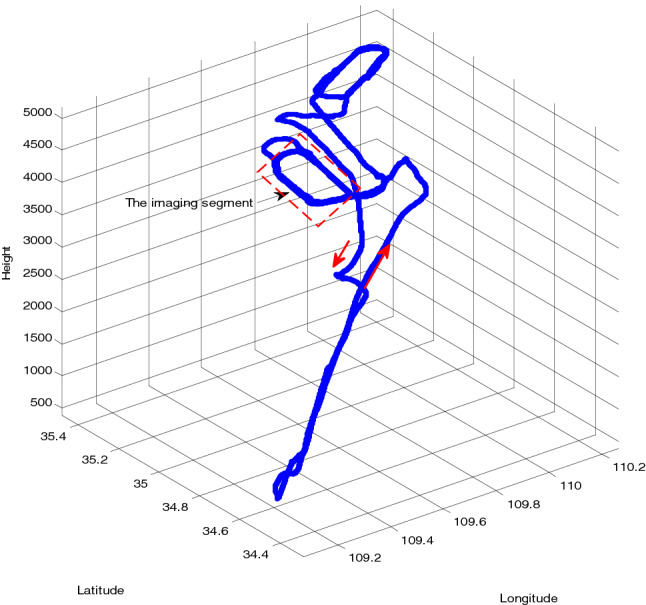
Flight trajectory.

To verify the practicability of the 45-dimensional high-order error model established in this paper, DPOS and radar loads are carried out in flight experiments. The equipment and flight path are shown in Figs. 9 and 10, respectively.

**Figure 11:**
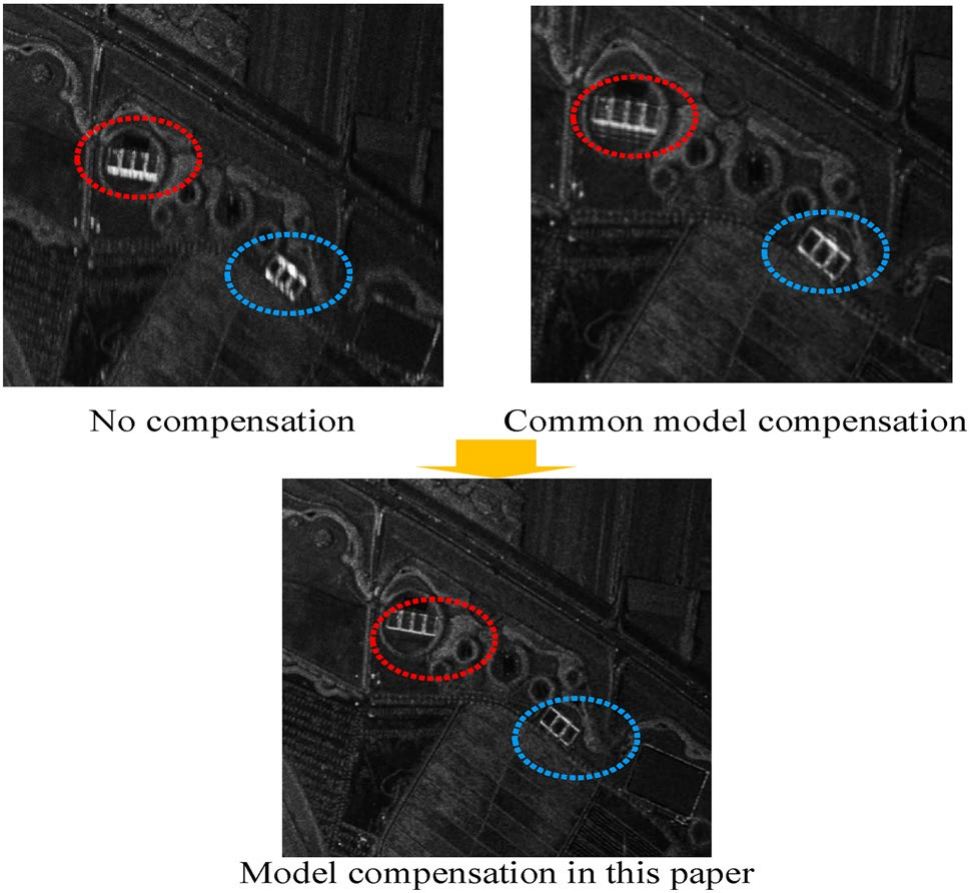
Contrast diagram of radar imaging precision.

Table 4 shows that the accuracy of latitude, longitude and height improved by 52.6%, 66.6%, and 30%, respectively, and the accuracy of azimuth angle, pitch angle and roll angle increased by 52.1%, 50.0%, and 57.1%, respectively. After using the model for motion compensation, the imaging precision is obviously improved. From Fig. 11, we can see that the imaging precision becomes higher after the use of the new mode.

**Table 4:** The relative navigation errors for the imaging segment (STD).

Standard deviation (STD)	Traditional error model	Error model in this paper
**Attitude error (°)**		
Azimuth	0.023	0.011
Pitch	0.008	0.004
Roll	0.007	0.003
**Position error (m)**		
Latitude	0.806	0.382
Longitude	0.915	0.305
Height	0.060	0.042

## Conclusions

In this work, high-precision and high-order transfer alignment models of DPOS in aerial remote sensing systems are proposed and verified by simulation experiments and real tests, respectively. The higher position and attitude accuracy of a slave POS can be obtained using the proposed high-order transfer alignment model based on the matching algorithm “position-velocity-attitude”. The effectiveness of the transfer models is verified by simulation experiments and vehicle and flight tests, which provide powerful theoretical reference for the engineering practice of a DPOS. In the future, the algorithm is expected to be implemented in a practical flight experiment based on the flexible long baseline and to enhance the development of aerial remote sensing technology.
